# Treatment of partial rotator cuff lesions is associated with a higher frequency of post-operative shoulder stiffness. A prospective investigation on the role of surgery-related risk factors for this complication

**DOI:** 10.1007/s00402-021-04285-1

**Published:** 2021-12-14

**Authors:** Davide Cucchi, Alessandra Menon, Sonia Maggi, Francesca Maria Feroldi, Annalisa De Silvestri, Max Julian Friedrich, Matthias Dominik Wimmer, Pietro Simone Randelli

**Affiliations:** 1 Department of Orthopaedics and Trauma Surgery, Universitätsklinikum Bonn, Venusberg-Campus 1, 53127 Bonn, Germany; 2 Laboratory of Applied Biomechanics, Department of Biomedical Sciences for Health, Università degli Studi di Milano, Via Mangiagalli 31, 20133 Milan, Italy; 3 U.O.C. 1° Clinica Ortopedica, ASST Centro Specialistico Ortopedico Traumatologico Gaetano Pini-CTO, Piazza Cardinal Ferrari 1, 20122 Milan, Italy; 4 Università degli Studi di Milano, Via Mangiagalli 31, 20133 Milan, Italy; 5 Biometry and Clinical Epidemiology, Scientific Direction, IRCCS San Matteo Hospital Foundation, Pavia, Italy; 6 Research Center for Adult and Pediatric Rheumatic Diseases (RECAP-RD), Department of Biomedical Sciences for Health, Università degli Studi di Milano, Via Mangiagalli 31, 20133 Milan, Italy

**Keywords:** Rotator cuff repair, Shoulder stiffness, Frozen shoulder, Adhesive capsulitis, Shoulder, Arthroscopy, Partial tear

## Abstract

**Purpose:**

Post-operative shoulder stiffness (SS) is a common complication after arthroscopic rotator cuff (RC) repair. The aim of this prospective study is to evaluate the role of surgical risk factors in the development of this complication, with special focus on the characteristics of the RC tears.

**Methods:**

Two-hundred and twenty patients who underwent arthroscopic RC repair for degenerative posterosuperior RC tears were included. Surgery-related risk factors for development of post-operative SS belonging to the following five categories were documented and analyzed: previous surgery, RC tear characteristics, hardware and repair type, concomitant procedures, time and duration of surgery. The incidence of post-operative SS was evaluated according to the criteria described by Brislin and colleagues.

**Results:**

The incidence of post-operative SS was 8.64%. The treatment of partial lesions by tear completion and repair technique was significantly associated with development of post-operative SS (*p* = 0.0083, *pc* = 0.04). A multivariate analysis revealed that treatment of partial lesions in patients younger than 60 years was associated to a higher risk of developing post-operative SS (*p* = 0.007). Previously known pre-operative risk factors such as female sex and younger age were confirmed. No other significant associations were documented.

**Conclusion:**

The treatment of partial lesions of the RC may lead to a higher risk of post-operative SS than the treatment of complete lesions, in particular in patients younger than 60 years. Possible explanations of this finding are the increased release of pro-inflammatory cytokines caused by the additional surgical trauma needed to complete the lesion and the different pain perception of the subgroup of patients who require surgical treatment already for partial tears.

**Evidence:**

A higher risk of post-operative SS should be expected after tear completion and repair of partial lesions, especially in young patients. Appropriate pre-operative counseling and post-operative rehabilitation should be considered when approaching this subgroup of RC tears.

**Level of evidence:**

Prognostic study, level II.

**Supplementary Information:**

The online version contains supplementary material available at 10.1007/s00402-021-04285-1.

## Introduction

Rotator cuff (RC) tears are one of the most common causes for shoulder pain and disability among the general population [1, 2]. With the growing age of the population and the increasing number of athletically active elderly, RC tears have required a larger number of surgical repairs. In the last decades, we have witnessed an important improvement of arthroscopic surgery and nowadays shoulder arthroscopy is considered the gold standard for RC tears repair, because it is safe, effective and with a success rate comparable to open surgery also at long-term follow-up [3–7]. Shoulder stiffness (SS) is a condition characterized by a painful reduction of both active and passive range of motion (ROM) and it is known to be a frequent complication of arthroscopic RC repair, which is described with variable incidence ranging between 1.5 and 32.7% [8, 9]. Different theories have been formulated to understand the mechanism behind post-operative SS, but the exact etiology has not been clarified yet. A surgery-related pro-inflammatory cytokine cascade appears likely to trigger fibrotic changes into the capsule and subsequent capsular contracture, fibroblast phenotypic shift and collagen deposition leading to adhesions [2, 8, 10–13].

Previous studies already investigated the role of surgery-related risk factors, in particular focusing on tear size, number of involved tendons, presence of additional lesions and surgical technique. However, no consensus has been obtained on the role of RC tear characteristics, with some authors claiming that partial tears and small sized tears are in strong correlation with the onset of post-operative SS, while other documenting an association between larger tears and SS [2, 3, 9, 14]. Furthermore, details on repair type and presence or treatment of associated conditions have only been scarcely investigated [3, 9, 15]. The goal of this study is to evaluate the incidence of post-operative SS and to investigate the role of five classes of surgery-related risk factors in the development of this complication, with special attention to the role of RC tear characteristics. The study hypothesis was that the treatment of partial and complete RC tears leads to a different risk of developing post-operative SS.

## Materials and methods

Patients referring to our institution to undergo arthroscopic RC repair for degenerative posterosuperior RC tears were assessed for eligibility. Patients with acute traumatic RC tears, shoulder instability, or presence of unequivocally diagnosed concomitant disorders of the shoulder, including fractures, glenohumeral arthritis, osteonecrosis or infection were excluded. Patients with clinical signs of pre-operative shoulder stiffness were excluded. Patients with isolated subscapularis tears and isolated bicipital pathology were also excluded.

All surgeries were performed under sedation and brachial plexus block by a single surgeon with extensive experience in shoulder arthroscopy. The patient was positioned in lateral decubitus, with a traction device keeping the upper limb kept at approximately 30° of flexion and 30° of abduction. Diagnostic arthroscopy was performed from standard posterior, midglenoid and lateral portals; the size of the tear was classified according to the Southern California Orthopaedic Institute (SCOI) classification [16] and the presence of intra-articular pathology was documented.

Either a standard single-row repair with titanium suture anchors (Super Revo^®^ FT and ThRevo^®^ FT Suture Anchors, Conmed, Utica, NY, USA) or a transosseous repair technique (Arthrotunneler™, Tornier, Bloomington, MN, USA) were used to address complete RC lesions, according to the surgeon’s preference. All partial lesions were completed and then treated with a single-row repair (Tear Completion and Repair technique, TCR). Acromioplasty was performed with Sampson’s cutting block technique in patients with type 2 or 3 acromial morphology according to Bigliani’s classification [17]. All the patients were operated by a single surgeon (P.S.R.). During and after surgery, information on five classes of intra-operative risk factors (Table 1) was collected by two examiners not involved in the tendon repair procedure and entered into a spreadsheet for analysis (F.M.F., S.M.).

**Table 1:** List of the surgery-related risk factors evaluated in the study, grouped into five categories

Previous surgery	Open surgery
	Arthroscopic surgery
RC tear characteristics	Size of the tear (SCOI classification)
	Associated subscapularis lesion
Hardware	Hardware type (metal or all-suture)
	Number of bone drills
Associated procedures	LHB procedures
	Subscapularis procedures
	Acromioplasty
	Associated capsular procedures
	Associated procedures on the acromioclavicular joint
	Biological augmentation
OR-Time	Surgical time
	Position in the OR-list

*OR* operating room, *SCOI* Southern California Orthopaedic Institute

The same rehabilitation protocol was adopted for all patients, regardless of lesion characteristics and repair technique used. Immediately after surgery, the shoulder was placed in a sling (Ultrasling II; Don Joy, Carlsbad, CA, USA). The day after surgery, patients were discharged with the recommendation to wear the sling all day long for 28 days (removal was allowed to perform personal hygiene and to eat) and instruction to perform early self-assisted light passive ROM exercises and exercises to mobilize the scapulothoracic joint, the elbow and the hand. Beginning from the 29th post-operative day, formal passive rehabilitation was initiated, under guidance of a dedicated physical therapist. Goals of this phase were full ROM recovery and, as soon as a satisfactory passive ROM was reached, begin of active training. The third phase of the rehabilitation, centered on regaining full muscle strength, started at end of the second month.

Post-operative SS was evaluated during routine post-operative follow-up visits and defined as the persistence of a motion deficit in passive external rotation (with the arm at the side: less than 10°; with the arm in 90° abduction: less than 30°), or in passive forward flexion (less than 100°) for at least 90 days post-operatively, as described by Brislin et al. [18].

Institutional review board approval was obtained by the local Ethical Committee (authorization number Fondazione IRCCS Ca' Granda Ospedale Maggiore Policlinico—Milano Area 2, Lombardia, Milan, n°123/2017, Milan, 27-02-2017).

### Statistical analysis

A power analysis prior to study begin indicated that a minimal sample size of 220 patients was sufficient to test the hypothesis that the treatment of partial and complete RC tears leads to a different risk of developing post-operative SS (chi-square test, *α* 0.05, *β* 0.8), assuming a 10% of post-operative SS and a difference of 30% of partial/complete RC between groups (development or not of SS).

Statistical analysis (A.D.S., A.M.) was performed using GraphPad Prism v 6.0 software (GraphPad Software Inc.) and Stata 16.1 (StataCorp USA).

Prevalence of post-operative SS is presented with 95% confidence interval (95% CI). Continuous variables were expressed as the mean ± standard deviation (SD) or medians and first and third quartiles [Q1–Q3], as appropriate. The Shapiro–Wilk normality test was used to evaluate the normal distribution of the sample and, if the null hypothesis of this test could not be rejected, the non-parametric Mann–Whitney test (*U* test) was applied for the analysis of the samples. Variables with a Gaussian distribution were analyzed with Student’s *t* test.

Categorical variables are expressed in numbers of cases and frequencies; their differences were tested using with the chi-square test or Fisher’s exact test. For all analyses, the significance level was set at *p* value lower than 0.05; to evaluate the associations between intra-operative factors and development of post-operative SS, a post hoc Bonferroni correction was applied taking into account the number of independent tests performed (i.e., the five categories in the first column of Table 3). A multivariate analysis with an interaction model was performed to adjust the results of the univariate analysis considering age and gender of the included patients.

## Results

Three hundred and eighty-five consecutive patients with indication for arthroscopic shoulder surgery were considered for inclusion over a period of 30 months and 235 meeting inclusion criteria were enrolled. Complete surgical and follow-up data for 220 patients were collected. For the remaining 15 patients, no follow-up data were available due to lack of compliance with the study protocol requirements.

**Fig. 1:**
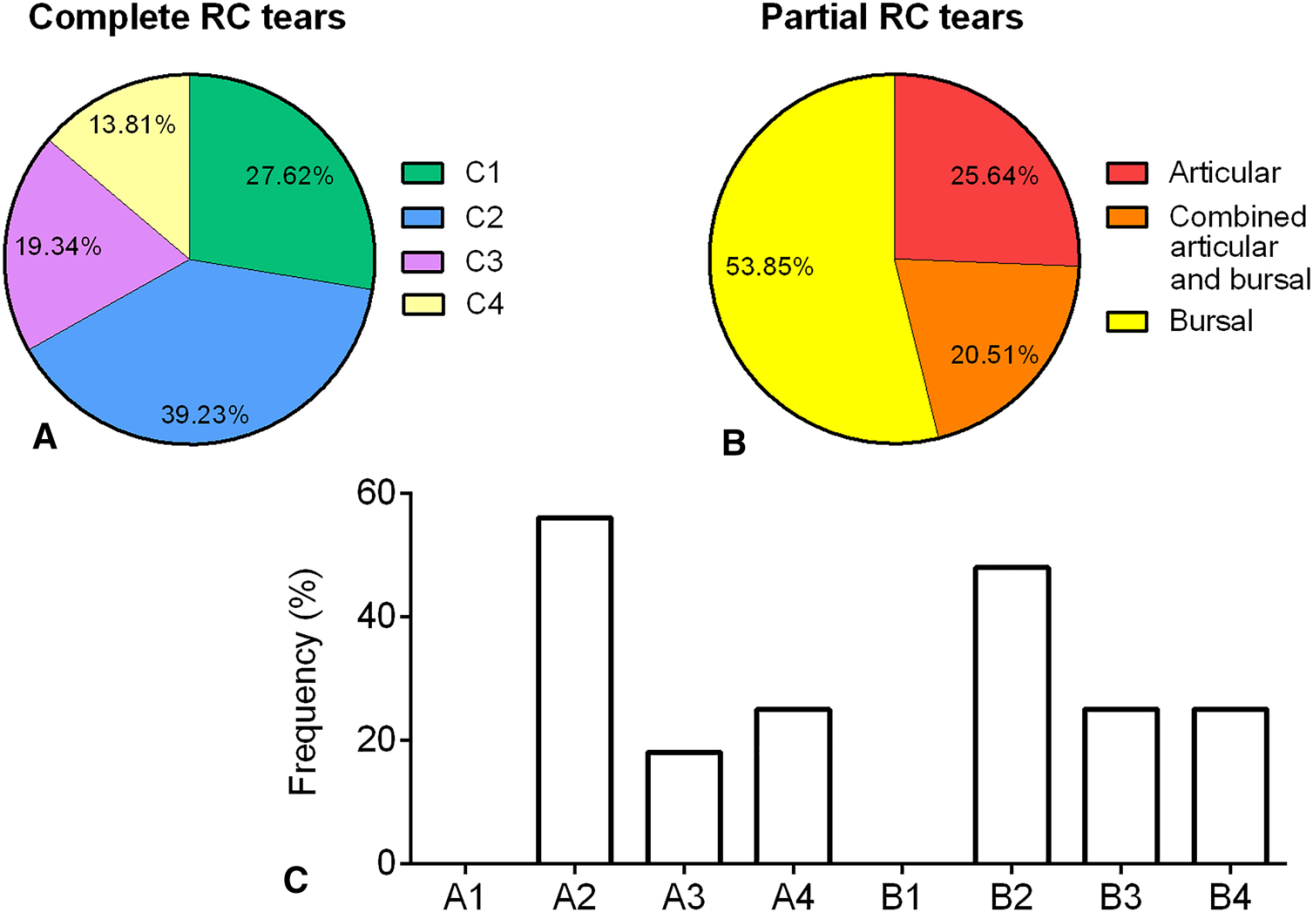
Distribution of rotator cuff (RC) tears across the study population, according to the SCOI classification. **A** distribution of complete RC tears; **B** distribution of partial RC tears, grouped as macrocategories “articular”, “bursal” and “combined articular and bursal”; **C** distribution of partial RC tears, grouped for tear types, according to the Southern California Orthopaedic Institute (SCOI) classification; since in some tears, articular and bursal lesions coexisted, the sum of all columns exceeds 100%

Patients’ demographics are shown in Table 2 and tears distribution according to the SCOI classification is illustrated in Fig. 1.

**Table 2:** Patient’s demographics

Group	Overall	Post-operative SS^+^	Post-operative SS ^−^	*p* value
Age (years)	59.70 (± 9.64)	53.31 (± 9.81)	60.31 (± 9.42)	**0.0023**
BMI (kg/m^2^)	25.66 (± 3.74)	24.32 (± 4.07)	25.78 (± 3.69)	0.1030 (n.s.)
Gender (F/M ratio)	0.52/0.48	0.89/0.11	0.48 /0.52	**0.0005**
Dominant side (L/R ratio)	0.04 /0.96	0.05 /0.95	0.04 /0.96	0.5634 (n.s.)
Surgery on dominant side (Y/N ratio)	0.62/0.38	0.73/0.27	0.64/0.36	0.0814 (n.s.)
Follow-up (months)	13.83 [8.85–20.92]	10.76 [8.86–14.23]	14.39 [8.85–21.35]	0.1202 (n.s.)

Bold values indicate statistically significant differences

Continuous variables were expressed as mean ± standard deviation (SD) or as median and interquartile range (first and third quartiles, Q1–Q3), as appropriate, while the dichotomous variables are expressed in numbers of cases and frequencies

*BMI* body mass index, *F/M* female/male, *L/R* left/right, *n.s.* not significant, *SS *^*+*^ post-operative shoulder stiffness, *SS *^*−*^ no post-operative shoulder stiffness, *Y/N* yes/no

A post-operative SS developed in 19 patients, representing an incidence of 8.64% [95% CI 5.3–13.2]. A significant dominance of younger patients (*p* = 0.0041) and females (*p* = 0.0005) was observed in the group who developed post-operative SS.

A significant association was found between the treatment of partial lesions and the development of post-operative SS (*p* = 0.0083; *pc* = 0.04). No other risk factors maintained statistical significance after Bonferroni correction. Specifically, presence of previous surgery, treatment of associated lesions, number of anchors or tunnel used, and surgical time did not appear to significantly affect the development of post-operative SS in the study cohort. The main results of the corrected univariate analysis are summarized in Table 3. Multivariate analysis confirmed that patients older than 60 years receiving treatment of complete lesions were protected against the development post-operative SS as compared to younger ones with partial tears (OR 0.161; 95% CI 0.042–0.614, *p* = 0.007).

**Table 3:** Summary of the main results for the study population

	Overall	Post-operative SS^+^	Post-operative SS^−^	*p* value
**Previous surgery**				
Open (Y/N ratio)	0.04/0.96	0.05/0.95	0.04/0.96	0.6027 (n.s.)
Arthroscopic (Y/N ratio)	0.09/0.91	0.21/0.79	0.08/0.92	0.0916 (n.s.)
**RC tear characteristics**				
Partial (Y/N ratio)	0.18/0.82	0.42/0.58	0.15/0.85	**0.0083 (0.04)**
C1 (Y/N ratio)	0.23/0.77	0.16/0.84	0.23/0.77	0.5753 (n.s.)
C2 (Y/N ratio)	0.32/0.68	0.32/0.68	0.32/0.68	1.0000 (n.s.)
C3–C4 (Y/N ratio)	0.27/0.77	0.10/0.90	0.29/0.71	0.1082 (n.s.**)**
SSc tear (Y/N ratio)	0.17/0.87	0/1	0.18/0.82	0.0490 (n.s.)
**Hardware**				
Type (metal/all-suture ratio)	0.92/0.08	0.89/0.11	0.92/0.08	0.6465 (n.s.)
No. bone drills	1.00 [1.00–2.00]	1.00 [1.00–1.00]	1.00 [1.00–2.00]	0.0202 (n.s.)
**Associated procedures**				
LHB (Y/N ratio)	0.71/0.29	0.58/0.42	0.72/0.28	0.1942 (n.s.)
SSc repair (Y/N ratio)	0.14/0.86	0/1	0.15/0.85	0.0828 (n.s.)
Acromioplasty (Y/N ratio)	0.96/0.04	0.89/0.11	0.97/0.03	0.1447 (n.s.)
Capsule (Y/N ratio)	0.04/0.96	0.05/0.95	0.04/0.96	0.5634 (n.s.)
ACJ (Y/N ratio)	0.04/0.96	0.10/0.90	0.03/0.97	0.1447 (n.s.)
Biologics (Y/N ratio)	0.04/0.96	0.10/0.90	0.04/0.96	0.2096 (n.s.)
**OR time**				
Surgical time	60.00 [50.00–80.00]	45.00 [40.00–75.00]	65.00 [50.00–80.00]	0.0708 (n.s.)
OR Position (first 3/last 3 ratio)	0.52/0.48	0.58/0.42	0.51/0.49	0.6370 (n.s.)

Bold values indicate statistically significant differences

Continuous variables were expressed as mean ± standard deviation (SD) or as median and interquartile range (first and third quartiles, Q1–Q3), as appropriate, while the dichotomous variables are expressed in numbers of cases and frequencies. Dichotomous variables are expressed in numbers of cases and frequencies. After Bonferroni correction, statistical significance was set at a level of 0.01

*ACJ* acromioclavicular joint, *LHB* long head of the biceps, *No*. number, *n.s.* not significant, *OR* operating room, *RC* rotator cuff, *SS*^*+*^ post-operative shoulder stiffness, *SS*^*−*^ no post-operative shoulder stiffness, *SSc* Subscapularis, *Y/N* yes/no

## Discussion

The main finding of this study is that the risk of developing post-operative SS is increased in young patients undergoing arthroscopic RC repair for the treatment of partial lesions with a TCR technique. SS is a condition of restriction in active and passive glenohumeral ROM, which can arise spontaneously or as consequence of a known cause, for which numerous risk factors have been described, but the exact etiology of which is not fully explained yet [2, 19, 20]. Post-operative SS is a subgroup of secondary SS, arising after surgical interventions around the shoulder joint and being probably related to the development of adhesion within soft tissue layers and capsular contractures [8]. Although the lack of consensus over the definition of “post-operative SS” leads to inhomogeneous reporting of this complication [8, 21] it is well known and recognized as a frequent undesired event after RC repair, with an incidence range from 1.5 to 11.1% [22] with the currently largest series reporting an incidence of 4.9% [3]. Our results in term of incidence of post-operative SS fall within the range of previously published series [3, 9, 14, 22–24]. As compared to the currently largest available series reporting on this complication, the incidence in the current cohort is slightly higher (4.9% according to Huberty et al., 8.64% in our series), possibly due to the stricter and more objective definition of SS used in our study, less prone to ruling out satisfied stiff patients[3]. In their series, Huberty et al. also observed that patients with single-tendon tears, smaller tears, and partial articular-sided tendon avulsion lesions were more likely to develop post-operative SS than those with full-thickness tears, larger in size, and/or involving more tendons, highlighting the relevance of surgery-related factors in the development of post-operative SS [3]. Similarly, Peters et al. measured an inferior ROM in abduction and external rotation and documented greater difficulty with reaching behind the back and with overhead activities six months after TCR of partial-thickness tears as compared to repair of full-thickness ones, however without obtaining a statistical significance in their results [14]. A recent clinical registry study by Audigé et al. also documented a similar rate of post-operative SS and identified partial tears treatment as a risk factor for developing post-operative SS [25]. Our study could also confirm that treatment of partial RC lesions with TCR is a risk factor for the development of post-operative SS. This finding can be explained according to three different causal clusters: mechanical, biological, and psychological factors.

Although generally believed to achieve uncomplicated complete healing, partial RC tears develop in a setting of poor tendon quality, which indeed leads to a substantial failure rate [14, 26]. As a possible explanation to this finding, Chung et al. described a higher grades of tendinosis in partial-thickness lesions than in full-thickness ones [27]. Such difference in tendon quality could derive from the tension mismatch between the layers of the partially torn tendon (which cease after full-thickness tendon tear), leading to microtears and tendinosis in a higher frequency in partial than in complete tears as well as from microvascular changes [28, 29]. Tendinosis can, hence, cause alteration of tendon mechanical properties and local inflammatory response, potentially negatively affecting tissue healing and rehabilitation [27, 29]. The biological response after TCR of partial RC tears is a further possible explanation to the higher rate of post-operative SS encountered in patient undergoing treatment of partial tears. As any additional surgical trauma, tear completion can provoke a more intense release of pro-inflammatory cytokines [30–32]. Release of pro-inflammatory cytokines, such as IL-1, IL-6 and TNFα, is one of the main events in the inflammatory cascade after surgical trauma, correlating to its extent [31–35]. These same cytokines play major role in dysregulation of the balance between matrix metalloproteinases and their inhibitors and in inducing phenotypic shift from fibroblasts to myofibroblasts, which are key events in the development of SS [10, 36, 37]. Nevertheless, the incidence of post-operative SS encountered in our series appears higher than previously published reports [38–40], with the exception of the series by Peters et al. [14] and Seo et al. [9]; however, different populations were investigated across those studies and variable goals, SS definitions and sample size were chosen, which makes objective comparison of the results difficult.

A final element possibly contributing to the higher incidence of post-operative SS in patients undergoing treatment of partial RC tears is pain perception: the subgroup of patients who require treatment for partial tears may have a tendency to localize shoulder pain in a more severe way: on one side, this implies requiring surgery already with smaller tears and, on the other side, the possibility of developing more a painful complication, such as SS more frequently. Studies on pain perception before and after shoulder surgery are limited and none explored the possible relation between the size of the RC tear and the level of perceived pain perceive in case of SS. Candela et al. investigated SS-related pain and its distribution: no correlation was reported between level of pain and age or affected side, but between female sex and higher grade of perceived pain. The authors suggest that a different nociceptive pain processing determined by sexual hormones leading to a higher central pain sensitization in females could explain these results [41]. Psychological factors, such as anxiety and depression, are also known to affect the grade of pain perception, symptoms duration and disability [42, 43]. In a recent study on patients undergoing arthroscopic RC repair, Thorpe et al. [44] evaluated cognitive psychologic factors, such as catastrophizing, negative pain belief and low pain self-efficacy, suggesting they could all be predictive of worse functional outcomes. On the other hand, George et al. [35] documented interactions between genetics and psychological factor affecting pain perception, catastrophizing and depression, suggesting that polymorphisms in KCNS1 (Potassium Voltage-Gated Channel Modifier Subfamily S Member 1) and ADRB2 (Adrenoceptor Beta 2) could be useful to predict pre-operatively shoulder disability and pain duration after surgery.

Numerous other risk factors have been described in relation to the occurrence of SS, including pre-, intra- and post-operative factors. In the last years, particular interest has been dedicated to the pre-operative risk factors, adding to the well-known ones such as diabetes mellitus and thyroid disease newer ones, like disorders of lipid metabolism and overweight, vitamin D deficiency, gastroesophageal diseases, genetic polymorphisms (e.g., IL-6 and MMP-3) and increased inflammatory changes in the shoulder joint [2, 9, 24, 25, 37, 45–48]. Considered the growing number of possible pre-operative risk factors for SS and the need of specific investigations to assess the presence of many of them, the authors decided to document and include in this study on the role of surgery-related risk factors only age, gender and body mass index, considering the first two also in the multivariate analysis.

The results of our study sharply contrast with those presented by Seo et al. [9], who retrospectively analyzed 119 patients and reported a much higher rate of post-operative SS (32.7%) with a higher frequency of SS after treatment of complete rather than partial tears. However, as the authors acknowledge, patient selection including those suffering from traumatic RC tears may strongly influence the results, leading to a higher rate of post-operative SS (42.9% in the trauma subgroup) and a shift in the distribution of SS towards complete tears. Traumatic tears are, in facts, most frequently complete [49, 50] and occur often in younger patients, which are more prone to develop post-operative SS than older ones [24]. Furthermore, if surgical treatment is performed shortly after injury, as usually occurs [50], the surgery-related release of pro-inflammatory cytokines may sum up to the inflammatory response generated by the injury itself, leading to a markedly different initial intra-articular environment as compared to the treatment of degenerative tears [30–32].

The strengths of this study are its prospective design, the strict inclusion criteria excluding confounding aspects such as traumatic RC tears and the use of objective clinical criteria to define SS [18]. Nevertheless, some limitations are present: first, the study was primarily focused on investigating the relevance of surgery-related factors in determining post-operative SS as single study outcome and both demographic pre-operative data and variables regarding the rehabilitation phase were not included in statistical analysis. Second, all surgeries were conducted by single, a high-volume, shoulder surgeon: the results of this study may not be applicable to a less experienced or lower volume surgeon, since surgical tissue traumatism, which may influence the inflammatory response after the procedure, can be dependent on the surgeon’s experience. Furthermore, the study was focused on the detection of surgery-related risk factors, and SS treatment protocols and results were not part of the research outcomes: patients diagnosed with SS were recommended to reduce pain-generating rehabilitation exercises, physiotherapist-assisted mobilization and stretching were encouraged and cortisone therapy was initiated, either as up to three repeated injections of 40 mg Triamcinolone acetonide or as oral therapy with Methylprednisolone [51–53]. Moreover, this study did not yet collect long-term follow-up data, thus not being able to show how post-operative SS affects mid- and long-term clinical outcomes and retear rates. Nevertheless previous studies documented a lower repair failure rate 6 months after surgery in patients who developed SS, suggesting that a rotator cuff repair is more likely to heal if this complication occur [54–56]. Furthermore, Millican et al. could evaluate up to nine years after surgery patients with post-operative SS, reporting a lower retear rate and a greater overall satisfaction by the final follow-up in patients experiencing post-operative SS; this suggests that SS after arthroscopic RC repair can have a protective role on the repair being either a sign of a strong biological healing response or an internal surrogate of a mechanical immobilization protecting the repair [54].

As final limitation, the lack of international recommendations on diagnostic criteria to define post-operative SS limits the possibility of a reliable comparison of our results with those from studies using different diagnostic criteria [21].

## Conclusions

The arthroscopic treatment of partial RC lesions by TCR is associated with a higher risk of post-operative SS than the treatment of complete lesions, in particular in patients younger than 60 years. Previously known pre-operative risk factors such as female sex and younger age were confirmed.

## Supplementary Information

Below is the link to the electronic supplementary material.

Supplementary file1 (DOCX 15 KB)
