# Supplementary material for: Treatment of partial rotator cuff lesions is associated with a higher frequency of post-operative shoulder stiffness. A prospective investigation on the role of surgery-related risk factors for this complication
Source: Arch Orthop Trauma Surg. 2021 Dec 14;142(11):3379–87. doi: 10.1007/s00402-021-04285-1 (PMC9522663; doi:10.1007/s00402-021-04285-1)
Supplement: Supplementary file 1 — Supplementary file1 (DOCX 15 KB) [file 402_2021_4285_MOESM1_ESM.docx]

**Supplementary Table E1: Patients’ demographics computed according to tear type.**

| **Group** | **Full tear** | **Partial tear** | ***p-value*** |
| --- | --- | --- | --- |
| **No. of patients** | **181** | **39** |  |
| **Age (years)** | 61.24 [55.59-68.35] | 52.13 (± 8.78) | ***<0.0001*** |
| **BMI (kg/m^2^)** | 25.96 (± 3.66) | 24.36 (± 4.01) | ***0.0157*** |
| **Gender (F/M ratio)** | 0.50/0.50 | 0.62 /0.38 | *0.2172 (n.s.)* |
| **Dominant side (L/R ratio)** | 0.04/0.96 | 0.03/0.97 | *1.0000 (n.s.)* |
| **Surgery on dominant side (Y/N ratio)** | 0.65/0.35 | 0.51/0.49 | *0.1453(n.s.)* |
| **Follow-up (months)** | 10.75 [8.88-18.92] | 14.08 [8.85-20.96] | *0.9461 (n.s.)* |

*Continuous variables were expressed as mean ± standard deviation (SD) or as median and first-third quartiles (Q1-Q3), as appropriate, while the dichotomous variables are expressed in numbers of cases and frequencies. BMI: body mass index; F/M: female/male; L/R: left/right; n.s.: not significant; Y/N: yes/no.*
